# Global, Regional, and National Burden of Falls Among Older Adults Aged 65 Years and Above: Secondary Data Analysis of the Global Burden of Disease Study 2021

**DOI:** 10.2196/73802

**Published:** 2026-04-10

**Authors:** Ze Zhang, Yingying Diao, Mingwang Fu, Wantong Han, Haoran Zhou, Ru Fan, Biyun Xu, Bingwei Chen

**Affiliations:** 1 Department of Epidemiology and Biostatistics, School of Public Health, Southeast University, Southeast University Dingjiaqiao Campus, No. 87 Dingjiaqiao, Gulou District, Nanjing, 210009, China, 86 83272562; 2 Medical Statistics and Analysis Center, Nanjing Drum Tower Hospital, Nanjing, China; 3 Key Laboratory of Environmental Medicine Engineering, Ministry of Education, School of Public Health, Southeast University, Nanjing, China

**Keywords:** falls, older adults, global burden of disease, mortality burden, disability burden, health inequality, projection

## Abstract

**Background:**

Falls are a leading cause of injury, disability, and death among older adults, posing significant public health challenges. However, comprehensive global analyses of fall-related burdens in older populations remain scarce.

**Objective:**

This study aimed to explore the patterns and distribution of the global, regional, and national burden of falls among adults aged 65 years and older.

**Methods:**

Data from the Global Burden of Disease study 2021 were used to assess the overall, disability, and mortality burden of falls among adults aged 65 years and older. Age-standardized rates of disability-adjusted life years (DALYs), years lived with disability (YLDs), and years of life lost were calculated to compare burdens across countries. Health inequalities were evaluated via the slope index of inequality and the concentration index. Frontier analysis identified optimal burden levels by sociodemographic index (SDI). Bayesian age-period-cohort models projected trends up to 2050.

**Results:**

DALY age-standardized rates showed a U-shaped distribution across SDI regions: lower-SDI countries faced higher mortality burdens, while higher-SDI countries had elevated disability burdens. Despite an increase in absolute overall burden inequality from 1990 to 2021, absolute inequalities in YLDs and years of life lost declined, with DALYs and YLDs exhibiting relatively more balanced distributions. Frontier analysis pinpointed countries with the greatest burden reduction potential. Projections suggest decreasing overall and mortality burdens by 2050 but rising disability burdens.

**Conclusions:**

Higher- and lower-SDI countries face distinct fall-related challenges. Reducing cross-national health inequalities and closing gaps between the observed burden and the optimal burden level achievable at a similar SDI level are critical. Despite projected declines in the overall burden (DALYs), the rising disability burden (YLDs) could present evolving challenges, potentially underscoring the importance of proactive preparedness.

## Introduction

Falls are one of the leading causes of injury, disability, and death among older adults, posing significant challenges to both public health and health care systems worldwide. As global populations age, the burden of falls is expected to increase, particularly among individuals aged 65 years and older. According to the Global Burden of Disease (GBD) study 2021, the age-standardized rate (ASR) of fall incidence was 6198.42 per 100,000 population in 2021, while the ASR of fall-related mortality was 78.36 per 100,000 population among older adults [1,2]. Furthermore, the World Health Organization reports that approximately 28% to 35% of older adults experience falls each year, resulting in outcomes ranging from minor injuries to severe fractures and mortality [3,4]. Beyond the immediate physical consequences, falls are linked to long-term disability, loss of independence, psychological trauma, and increased health care costs, making them a critical concern for both individuals and society at large [5,6].

In light of this growing issue, understanding the global, regional, and national burden of falls has become increasingly important. The GBD study 2021 offers a robust framework for quantifying the impact of diseases and injuries worldwide, including falls. While falls among older adults have been widely studied, many of the existing studies are limited in scope—either focusing on specific regions or countries or addressing only certain aspects of mental and physical effects [7-10]. A study based on the GBD study discussed the mortality rate of falls across 59 high-income and upper-middle–income countries [11]. However, significant gaps persist in comprehensively understanding the global-scale mortality and disability burdens across diverse populations and regions, as well as the underlying socioeconomic, environmental, and health care–related factors driving these disparities.

This study aimed to examine the global, regional, and national burden of falls among individuals aged 65 years and older using the latest data from the GBD study 2021. By analyzing variations in fall burdens across countries or regions, the research sought to identify current and future challenges across different development levels and geographic locations.

## Methods

### Study Data

This study was a secondary data analysis primarily using the GBD study 2021 database [1,2]. The GBD study 2021, led by the Institute for Health Metrics and Evaluation at the University of Washington, provides comprehensive global health data, covering 369 diseases, injuries, and risk factors. Data on the burden of falls from 1990 to 2021 were retrieved using the GBD Results Tool across 7 age groups: 65 to 69 years, 70 to 74 years, 75 to 79 years, 80 to 84 years, 85 to 89 years, 90 to 94 years, and 95 years and above [12]. Demographic data from 1990 to 2050 were acquired from the GBD study database and a projection of the population [13,14]. Additionally, the sociodemographic index (SDI) was accessed from the GBD study database to evaluate the development status of the included countries and territories [15].

### Definition

The disability-adjusted life years (DALYs) represent the total number of years lost due to ill-health, disability, or early death. This study selected DALYs as the measure of overall burden of falls, with years lived with disability (YLDs) measuring burden of disability and years of life lost (YLLs) measuring burden due to premature mortality. ASR is a statistical measure used to compare rates of health metrics (per 100,000 people) across different populations, adjusting for variations in age distribution. By standardizing the measures using the GBD study world population age standard, the rates of DALYs, YLDs, and YLLs become comparable across countries or regions. The SDI is a composite measure used to summarize a country or region’s sociodemographic development; it ranges from 0 to 1, where higher values indicate greater sociodemographic development [16,17].

### Statistical Analysis

Joinpoint regression analysis was performed using the Windows command-line (batch/callable) version of Joinpoint Regression Software (Surveillance Research Program, National Cancer Institute) to acquire the annual percentage change (APC) and average APC of ASRs [18-20]. This study calculated 2 key indexes of health inequality: the slope index of inequality (SII) and the concentration index, which reflect absolute and relative health inequities, respectively. The SII measures absolute inequality by quantifying the difference in indicator values between the most disadvantaged and most advantaged subgroups considering the overall population distribution. It is derived from a regression model that accounts for the cumulative distribution of the population. Larger SII values indicate greater disparities between these groups. The concentration index is a relative measure that assesses the concentration of a health indicator across subgroups. It ranges from −1 to 1, with 0 indicating no inequality. Positive values suggest concentration in advantaged groups, while negative values indicate concentration among disadvantaged groups. The further the concentration index is from 0, the greater the level of inequality [21]. Frontier analysis was conducted to identify the optimal burden levels that countries could achieve based on their corresponding development statuses [22]. A Spearman rank correlation analysis was conducted to evaluate the relationship between the burden of falls and aging-related indicators among older adults. Bayesian age-period-cohort models and integrated nested Laplace approximations using the R packages *BAPC* (version 0.0.36) and *INLA* (version 24.06.27; R Foundation for Statistical Computing) were used to project the future trends in the burden of falls among older adults [23,24].

### Ethical Considerations

This study was a secondary analysis of publicly available, deidentified, aggregated data from the GBD study 2021, used in accordance with the relevant data use terms. No additional ethics approval was sought for this study because it was based exclusively on publicly available, anonymized data and involved no direct contact with human participants. This study involved no collection of, or access to, identifiable personal information; therefore, additional informed consent was not required. All analyses were conducted using anonymized, publicly available data, ensuring privacy and confidentiality. No participants were recruited, and no compensation was provided.

## Results

### Spatiotemporal Patterns of Falls Among Older Adults

In the initial analysis, it was found that ASRs of mortality and incidence due to falls varied among countries with different SDIs (Figures S1-S3 in Multimedia Appendix 1 and Tables S1-S4 in Multimedia Appendix 2). Higher-SDI countries exhibited lower ASRs for mortality and higher ASRs for fall incidence, whereas lower-SDI countries exhibited more falls and higher mortality rates among older adults. To gain a comprehensive understanding, we used DALYs, YLDs, and YLLs as indicators to assess the overall burden, disability burden, and mortality burden of falls among individuals aged 65 years and above (Tables S5-S10 in Multimedia Appendix 2).

**Figure 1.**
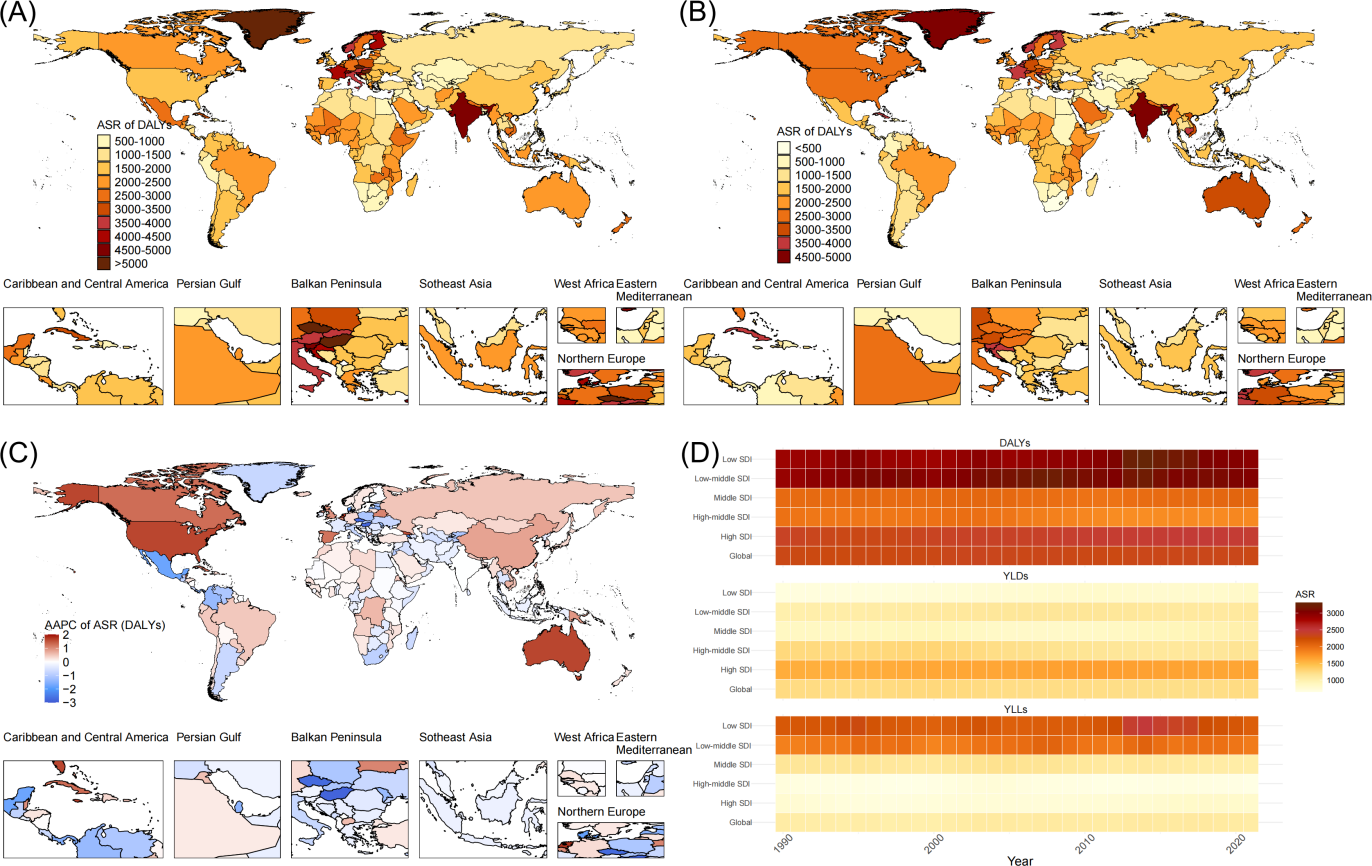
Spatiotemporal patterns of burden of falls among older adults: (A) age-standardized rate (ASR) of disability-adjusted life years (DALYs) in 1990; (B) ASR of DALYs in 2021; (C) average annual percentage change (AAPC) in the ASR of DALYs from 1990 to 2021; and (D) heat map of the ASR of DALYs, years lived with disability (YLDs), and years of life lost (YLLs) at the global and sociodemographic index (SDI) region levels in 2021.

From 1990 to 2021, the overall burden of falls among older adults remained consistently high in India, Australia, Europe, North America, and Africa (Figures 1A and 1B). Specifically, North America and Australia exhibited an increasing trend in their overall burden, while other regions maintained stable levels or experienced only minor increases (Figure 1C). Regarding disability and mortality burdens, regions with low and low-middle SDI exhibited higher mortality burdens, whereas high-SDI regions exhibited greater disability burdens. The overall burden of falls among older adults followed a U-shaped distribution, with middle- and high-middle–SDI regions experiencing the lowest burden and low-, low-middle–, and high-SDI regions facing heavier overall burdens (Figure 1D).

### Health Inequality Analysis

**Figure 2.**
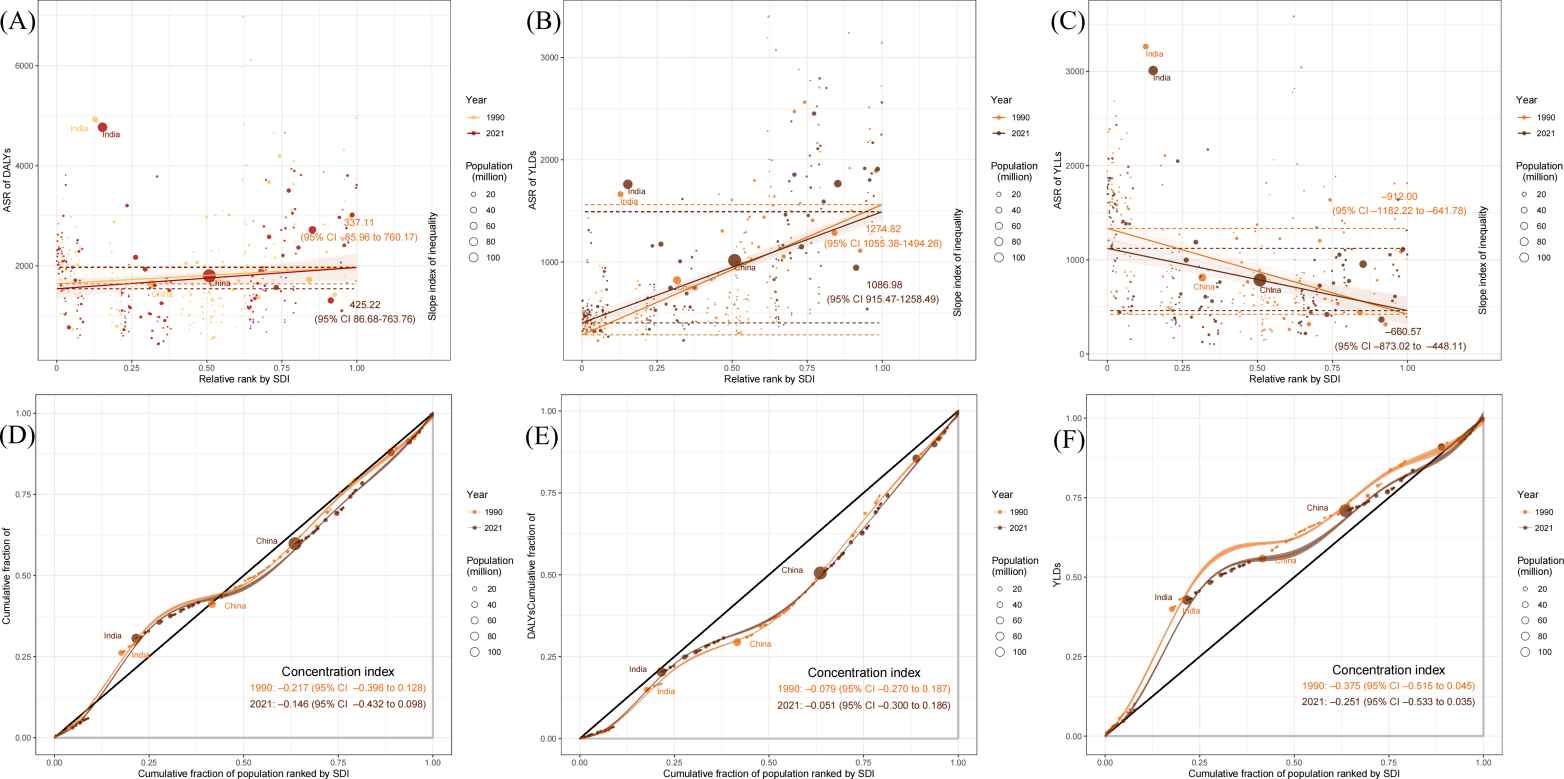
Health inequality analysis: (A-C) slope indexes of inequality for the age-standardized rates (ASRs) of disability-adjusted life years (DALYs), years lived with disability (YLDs), and years of life lost (YLLs) and (D-F) concentration indexes for DALYs, YLDs, and YLLs. CI: confidence interval. SDI: sociodemographic index.

The SII was used to quantify absolute differences in ASRs (rates) of burden indicators between the most disadvantaged and most advantaged countries. From 1990 to 2021, the SII for DALYs increased from 337.11 to 425.22, indicating a widening of absolute overall health inequality (Figure 2A). In contrast, the SII for YLDs decreased from 1274.84 to 1086.98, and the SII for YLLs decreased from −912.00 to −660.57, suggesting a reduction in the absolute inequality of both disability and mortality burdens (Figures 2B and 2C). Consistent with the patterns presented in Figure 1D, these metrics reveal distinct underlying drivers for disability and mortality: the positive SII for YLDs confirms that the disability burden was disproportionately concentrated in higher-SDI countries, while the negative SII for YLLs aligns with our finding that the mortality burden remained more pronounced in lower-SDI regions.

Distinct from the SII, which compares absolute inequality at the country level, the concentration index evaluates relative health inequality by analyzing the distribution of burden indicators (total numbers) across the global population ranked by SDI. Between 1990 and 2021, the concentration index for DALYs shifted from −0.217 to −0.146, indicating a reduction in relative inequality. For YLDs, the concentration index values remained close to 0 (−0.079; *P*=.50; and −0.051; *P*=.68, respectively), suggesting that the disability burden was distributed relatively evenly across the global population [25]. Meanwhile, the concentration index for YLLs increased from −0.375 to −0.251, reflecting a narrowing of relative inequality in mortality. Notably, the negative concentration indexes for both DALYs and YLLs indicate that, despite improvements over time, the overall and mortality burdens remained disproportionately concentrated in lower-SDI populations.

### Frontier Analysis

**Figure 3.**
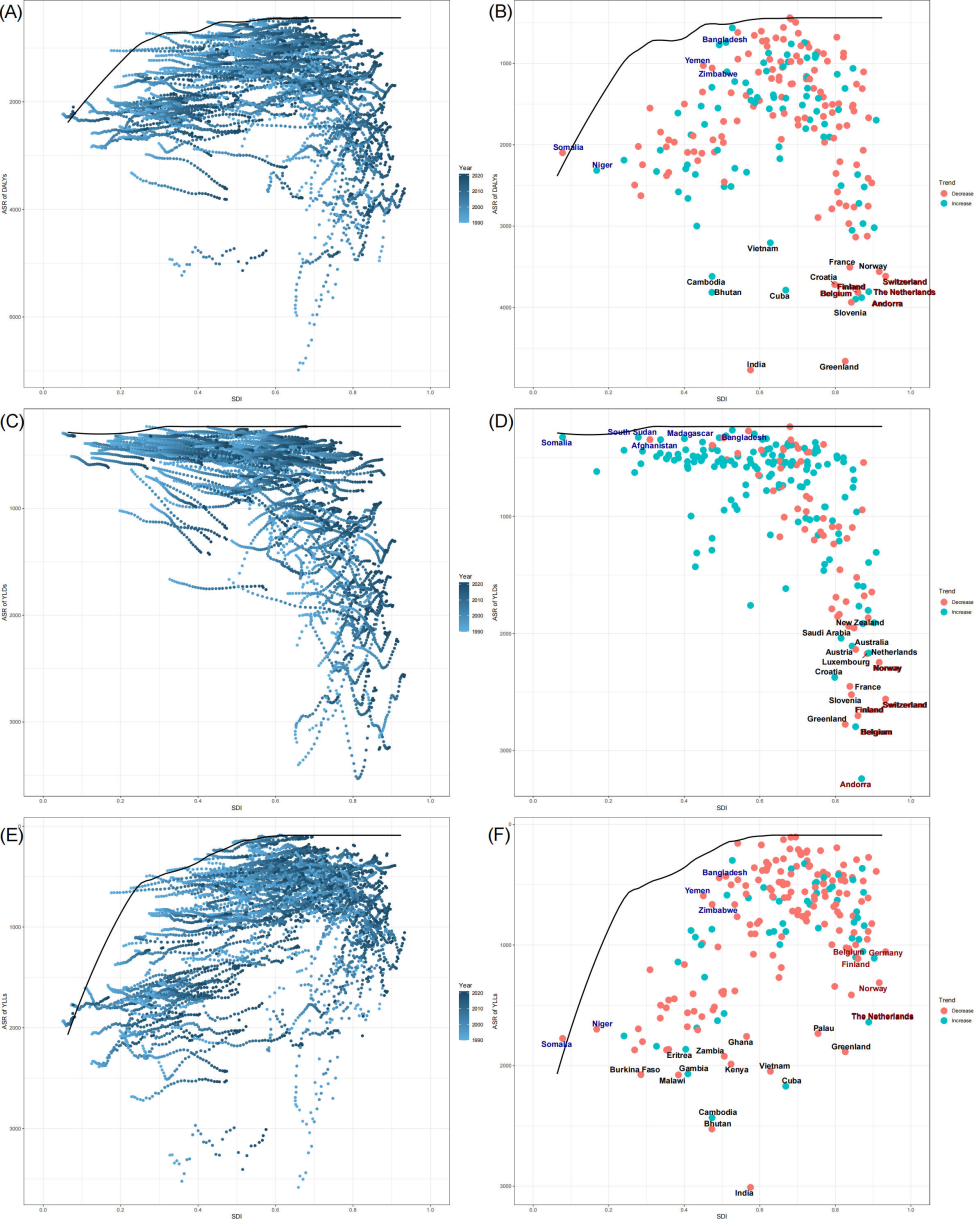
Frontier analysis based on sociodemographic index (SDI) and age-standardized rates (ASRs) of disability-adjusted life years (DALYs), years lived with disability (YLDs), and years of life lost (YLLs) in 204 countries and territories: (A-B) ideal ASRs of DALYs corresponding to the SDI, (C-D) ideal ASRs of YLDs corresponding to the SDI, and (E-F) ideal ASRs of YLLs corresponding to the SDI.

Frontier analysis was conducted to determine the lowest burden that could be potentially achieved based on development status. In Figures 3A, 3C, and 3E, the scatterplot illustrates the relationship between SDI and the ASRs of DALYs, YLDs, YLLs (the color change from light blue [1990] to dark blue [2021]), indicating a shift in their distribution over time. The black frontier line represents the efficiency frontier, which delineates the lowest possible burden of DALYs, YLDs, and YLLs for a given SDI level. The distance from the frontier represents the gap between the observed burden and the potentially achievable burden of falls in a given country or territory considering its SDI. For ASRs of DALYs, countries with both low and high SDIs experienced a higher burden, while those with middle SDI levels had the lowest DALYs. In addition, for ASRs of YLDs, countries with higher SDIs generally exhibited higher disability burdens. High-SDI countries showed a declining or stable trend, while low-SDI countries exhibited an increasing trend in YLDs. In contrast, for ASRs of YLLs, most curves showed a stable or slight decline, and countries with higher SDIs generally exhibited lower mortality burdens.

In Figures 3B, 3D, and 3F, the scatterplots show the relationship between SDI and the ASRs of DALYs, YLDs, and YLLs across different countries in 2021. The colors indicate the trend of ASRs between 1990 and 2021, where red dots represent countries with a decrease in ASRs, while blue dots indicate an increase. High-SDI countries (SDI>0.85) with the top 5 largest distances are marked in red. In contrast, among countries with an SDI below 0.50, those performing best are marked in blue. Additionally, the top 15 countries and territories that are furthest from the frontier are colored in black.

Among the ASRs of DALYs, the countries furthest from the frontier line include India, Greenland, Slovenia, Belgium, and Andorra. For ASRs of YLDs, the countries furthest from the frontier line include Andorra, Belgium, Greenland, Finland, and Switzerland. Regarding ASRs of YLLs, the countries furthest from the frontier line include India, Bhutan, Cambodia, Cuba, and Vietnam. The frontier fit line for ASRs of YLDs is flatter than those of other burden indicators, denoting that the ideal ASRs of YLDs vary little across SDI levels.

### Projections of Burden of Falls Among Older Adults

**Figure 4.**
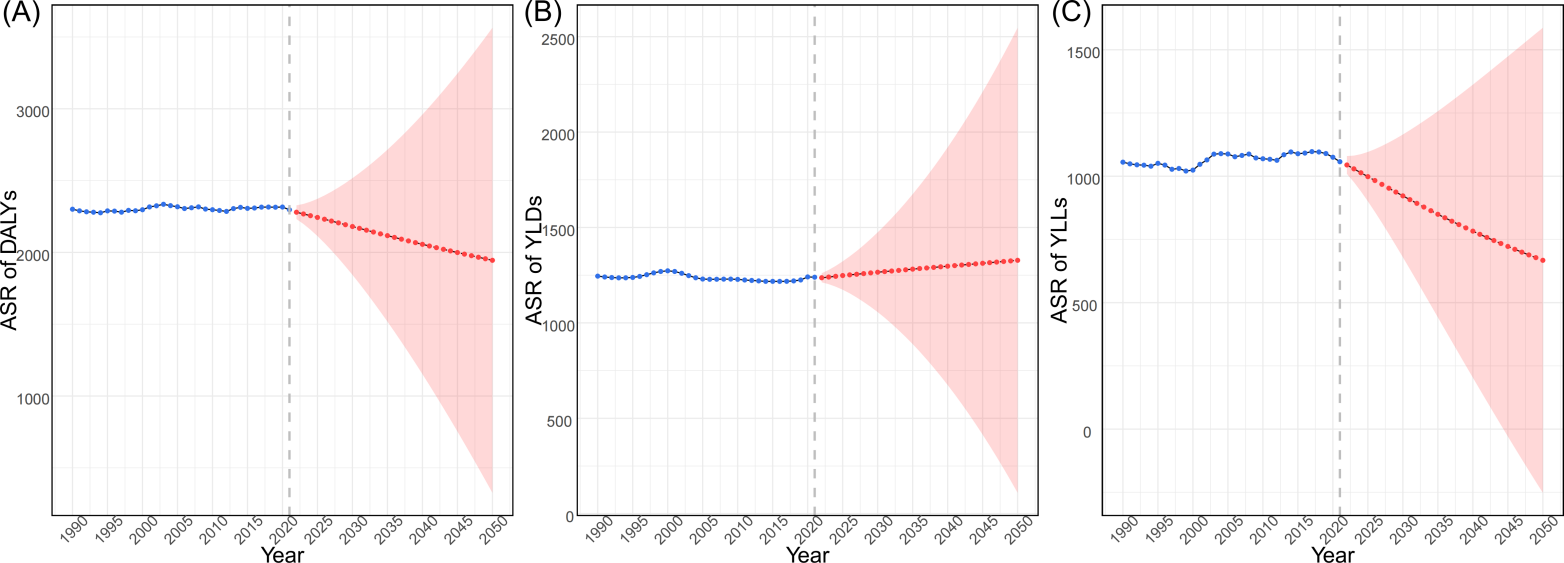
Projection of age-standardized rates (ASRs) of disability-adjusted life years (DALYs), years lived with disability (YLDs), and years of life lost (YLLs) up to 2050.

This study projected the global ASRs of DALYs, YLDs, and YLLs over the next 3 decades by integrating demographic forecasts from 2020 to 2050 using the Bayesian age-period-cohort model (Figure 4). The blue dots in the figure illustrate actual data from 1990 to 2021, and the red dots represent forecasts for the period spanning 2022 to 2050. Overall, the global burden exhibits a declining trend. However, the ASR of YLDs shows an upward trend, indicating that the disability burden may become the future challenge in terms of improving falls among older adults.

## Discussion

### Principal Findings

In this study, we used DALYs, YLDs, and YLLs to evaluate the overall, disability, and mortality burden of falls among individuals aged 65 years and above. APCs and average APCs of ASRs were calculated to provide insights into trends in burden from 1990 to 2021. Our findings demonstrate that the burden of falls exhibited distinct patterns according to development status. Specifically, YLDs (disability burden) were more prevalent in high-SDI regions, whereas YLLs (mortality burden) were more concentrated in low- and low-middle–SDI regions, reflecting a heavier mortality burden. Consequently, the overall burden (DALYs) followed a U-shaped distribution, with burdens being comparatively more severe at both development extremes.

The observed variations in fall-related burden across geographical regions appear to mirror the broader patterns observed across different levels of socioeconomic development. In high-SDI regions such as Australia, Europe, and North America, the high burdens are primarily driven by older population structures [26,27]. However, beyond demographics, environmental and lifestyle factors play a significant role. Higher rates of urbanization and sedentary lifestyles in these regions may contribute to age-related sarcopenia and impaired balance [28-30]. While infrastructure in high-income settings is generally superior, the urban built environment introduces unique hazards; complex public transportation systems, high-speed escalators, and dense pedestrian traffic can pose challenges for older adults with diminished reaction times [31-33]. Additionally, seasonal climatic factors significantly modulate this burden in high-latitude regions, where winter ice and snow create hazardous conditions that drive seasonal surges in fall-related fractures [34-36]. Furthermore, while health systems in these regions often demonstrate high proficiency in acute trauma surgery, potentially reducing short-term mortality, the elevated YLDs potentially point toward a relative gap in specialized geriatric rehabilitation [37-40]. This indicates that, although life-saving interventions are effective, the current infrastructure for long-term fall prevention and functional recovery might not yet fully keep pace with the needs of an increasing aging population [41-43].

In contrast, the burden observed in countries such as India and parts of the African region appears to reflect a dual pattern of persistent mortality alongside rising disability, potentially shaped by an interplay of systemic, environmental, and cultural factors [44-47]. Structurally, health system capacity may represent a significant challenge; limited emergency medical services and a scarcity of geriatric specialists are potentially associated with suboptimal clinical outcomes and higher case fatality rates following fall injuries [48]. Environmentally, natural topography and infrastructure limitations appear to play a notable role, particularly in rural settings. Older adults often navigate uneven, unpaved terrain and steep slopes, frequently without the aid of stabilized walkways or safety railings [49,50]. These risks may be further compounded by insufficient lighting in both communal and domestic spaces, which can hinder navigation for individuals with age-related visual impairments [51,52]. Additionally, domestic environments in these regions may not always feature age-friendly modifications such as grab bars or standardized step heights [53]. Culturally, while the traditional reliance on informal family caregiving is significant, access to the specialized medical and physiotherapeutic knowledge required for effective postfall rehabilitation may be limited [54-56]. Without professional guidance on secondary prevention, these informal care structures, despite their supportive intent, might correlate with challenges in preventing recurrent falls and managing long-term disability [57,58].

The observed epidemiological transition in fall-related burdens is further elucidated by the health inequality analysis, which reveals distinct patterns between absolute gaps (country-level rates) and relative distributions (population-level volume). Regarding absolute inequality, the positive SII for DALYs and YLDs indicates that higher-SDI countries bear a greater absolute burden per 100,000 population, particularly in terms of disability. This is likely attributable to the fact that higher-income countries possess more advanced medical and nursing resources, ensuring timely intervention and survival after a fall. While these resources successfully reduce the mortality burden (YLLs), the combination of survival following injury and a higher proportion of older individuals leads to a more pronounced disability burden. Conversely, the negative SII for YLLs confirms that mortality rates remain higher in lower-income regions, where limited medical infrastructure often results in higher fatality rates following falls. From a temporal perspective, the period from 1990 to 2021 witnessed a narrowing of the absolute gaps in both disability (YLDs) and mortality (YLLs) between the most advantaged and least advantaged countries. It was observed that the absolute gap in the overall burden (DALYs) expanded during this time frame; however, this divergence should be interpreted with caution. It appears to be linked to the reduction in age-standardized DALY rates in lower-SDI countries, suggesting that the gap may be widening due to unequal rates of improvement rather than worsening outcomes [59,60].

While absolute gaps indicate disparities in rates between the highest- and lowest-income countries, the analysis of relative inequality (concentration index) provides a complementary perspective by examining how the total burden (absolute number) is distributed across the global population ranked by SDI. Notably, a near-zero concentration index for YLDs suggests a relatively even distribution of disability, and the negative concentration indexes for DALYs and YLLs underscore a persistent health inequity. This implies that, despite higher disability rates in the highest-income countries, the global volume of mortality and overall burden remains predominantly concentrated among populations in lower-income regions. This concentration of burden appears to be driven by 2 primary factors: first, lower-SDI countries often have significantly larger population sizes, meaning that even a moderate incidence rate translates into a much larger absolute number of affected individuals, and second, the dominant contribution of mortality (YLLs) in disadvantaged regions is often exacerbated by inadequate emergency response and a lack of specialized geriatric rehabilitation. Therefore, while high-SDI countries are increasingly challenged by the long-term care of survivors, the most disadvantaged populations continue to carry the heaviest cumulative weight of the burden due to high fatality rates and large population volumes.

However, this global landscape has evolved favorably over the past 3 decades, as indicated by the narrowing concentration indexes for DALYs and YLLs between 1990 and 2021, suggesting a more balanced global distribution of the fall burden. This shift may be partly attributable to the socioeconomic advancements in populous emerging economies, most notably China and India. As these countries experienced steady increases in their SDI, their massive populations gradually shifted toward higher-SDI quintiles. This demographic-economic transition has played a pivotal role in reducing relative inequality. In summary, while absolute metrics (SII) highlight an increasing disability rate in high-SDI countries as a byproduct of aging and survival, relative indexes (concentration index) emphasize that global health efforts must remain focused on the persistent inequities in lower-income areas. Thus, addressing the fall-related burden requires a dual strategy: managing the rising disability in aging, high-income societies while simultaneously tackling the high mortality burden in disadvantaged populations.

The frontier analysis identified the ideal levels of burden that countries could achieve given their stage of development. Thus, countries positioned farther from the frontier have greater potential to enhance fall prevention among older adults. The analysis also highlights which countries excel in controlling the burden of falls and which fail to meet these ideal levels. Although one might expect higher-SDI countries to more effectively manage the burden of falls in older populations, the relatively flat frontier curve for YLDs against SDI suggests little difference in ideal burden of disability between lower- and higher-SDI countries.

Projections of the fall-related burden among older adults highlight potential public health challenges in the coming decades. Although the age-standardized overall burden (DALYs) is expected to decline, the ASR of YLDs is projected to increase, suggesting that the disability burden may pose an increasingly significant future challenge. To mitigate this anticipated shift toward a disability-predominant burden, forward-looking strategies must simultaneously address 3 critical dimensions: preserving intrinsic capacity, minimizing extrinsic hazards, and generalizing system capacity.

Specifically, prevention efforts may benefit from prioritizing the preservation and enhancement of intrinsic capacity, defined as the composite of an individual’s physical and mental reserves [61]. The integration of systematic screening approaches such as the World Health Organization’s integrated care for older people framework into primary health care systems could facilitate the early identification of deficits in functional ability [62,63]. Crucially, screening must be coupled with targeted interventions; identifying risk is only the first step. Comprehensive management should extend to personalized lifestyle guidance, encompassing nutritional optimization to combat sarcopenia, sleep hygiene to maintain cognitive alertness, and psychological support to alleviate the fear of falling [64-66]. Subsequent individualized exercise prescriptions, such as the Otago Exercise Program or tai chi, should be implemented to improve muscle strength and balance [67,68]. This proactive continuum aims to reverse physical frailty, thereby fortifying the individual’s intrinsic resilience against falls.

Complementing these active measures, policy efforts may benefit from prioritizing the reduction in extrinsic hazards through environmental modifications. Implementing universal design principles in urban planning, including barrier-free walkways and adequate street lighting, alongside subsidizing home modifications such as grab bars and nonslip flooring, is critical for supporting “aging-in-place” [69-71]. By systematically mitigating external environmental stressors, such measures can provide a passive safety buffer that operates independently of an individual’s fluctuating physical condition, thereby lowering the probability of injury in daily life.

Finally, the adoption of emerging technologies offers a potential solution to generalize and extend system capacity, bridging the workforce gap in geriatric care [72,73]. Wearable fall detection sensors and tele-rehabilitation platforms could provide continuous, unobtrusive monitoring and accessible home-based training [74-77]. These technologies hold the potential to democratize access to specialized resources, supporting the delivery of fall prevention and rehabilitation services and ensuring they are available even in resource-constrained or remote settings.

### Limitations

While this study provides a comprehensive global overview of fall-related burdens among older adults, several limitations warrant consideration. First, this research used a macrolevel analysis examining national data from 1990 to 2021; consequently, it did not involve in-depth clinical investigations or granular subnational analyses of individual countries. Localized heterogeneity, such as specific rural-urban disparities within a single country, unique cultural safety practices, or variations in regional infrastructure, may not be fully captured by national-level SDI metrics. Second, the findings are subject to the inherent limitations of the GBD study’s primary data sources. In many low-SDI regions, vital registration systems may be fragmented or inconsistent. This can lead to potential underreporting or the misclassification of fall-related injuries. However, to mitigate these disparities, the GBD study framework uses rigorous statistical modeling methods to calibrate data inconsistencies and fill gaps. While these advanced techniques significantly reduce bias and ensure global comparability, the estimates in data-sparse regions should still be interpreted with a degree of caution. Finally, while the frontier and inequality analyses provide robust insights into global patterns, they are based on aggregated statistical models. These models describe observed trends and performance gaps but do not account for sudden shifts in health policy or major global disruptions. Future research using more granular, local-level data would be beneficial to validate these national trends and further explore the specific sociocultural drivers of the fall-related burden.

### Conclusions

This study elucidates divergent patterns in the global burden of falls among older adults, consistent with an epidemiological transition: lower-SDI regions bear a predominant mortality burden (YLLs), while higher-SDI regions face an elevated disability burden (YLDs). Regarding health inequality, although the relative distribution of the overall burden (concentration index) showed signs of convergence during the study period, the absolute gap (SII) in ASRs between the most and least advantaged populations appears to persist. Furthermore, the frontier analysis indicated that potential exists for countries across the sociodemographic spectrum to further reduce their burden relative to achievable benchmarks.

Projections of the fall-related burden suggest that, while overall rates may decline, the disability burden (YLDs) is likely to pose an increasingly significant challenge in the coming decades. Collectively, these findings suggest that enhanced proactive preparedness could play a pivotal role in mitigating this anticipated future strain.

## Supplementary material

10.2196/73802 Multimedia Appendix 1 Spatiotemporal patterns of incidence and mortality and Joinpoint regression analyses of temporal trends in falls among adults aged 65 years and older.

10.2196/73802 Multimedia Appendix 2 Additional results on cross-national variations in fall-related mortality, incidence, disability-adjusted life years, years lived with disability, and years of life lost among adults aged 65 years and older by sociodemographic index level.

## Abbreviations

APC: annual percentage change
ASR: age-standardized rate
DALY: disability-adjusted life year
GBD: Global Burden of Disease
SDI: sociodemographic index
SII: slope index of inequality
YLD: year lived with disability
YLL: year of life lost
