# Supplementary material for: Global, Regional, and National Burden of Falls Among Older Adults Aged 65 Years and Above: Secondary Data Analysis of the Global Burden of Disease Study 2021
Source: JMIR Aging. 2026 Apr 10;9:e73802. doi: 10.2196/73802 (PMC13068194; doi:10.2196/73802)
Supplement: Multimedia Appendix 1 [file aging-v9-e73802-s001.docx]

| 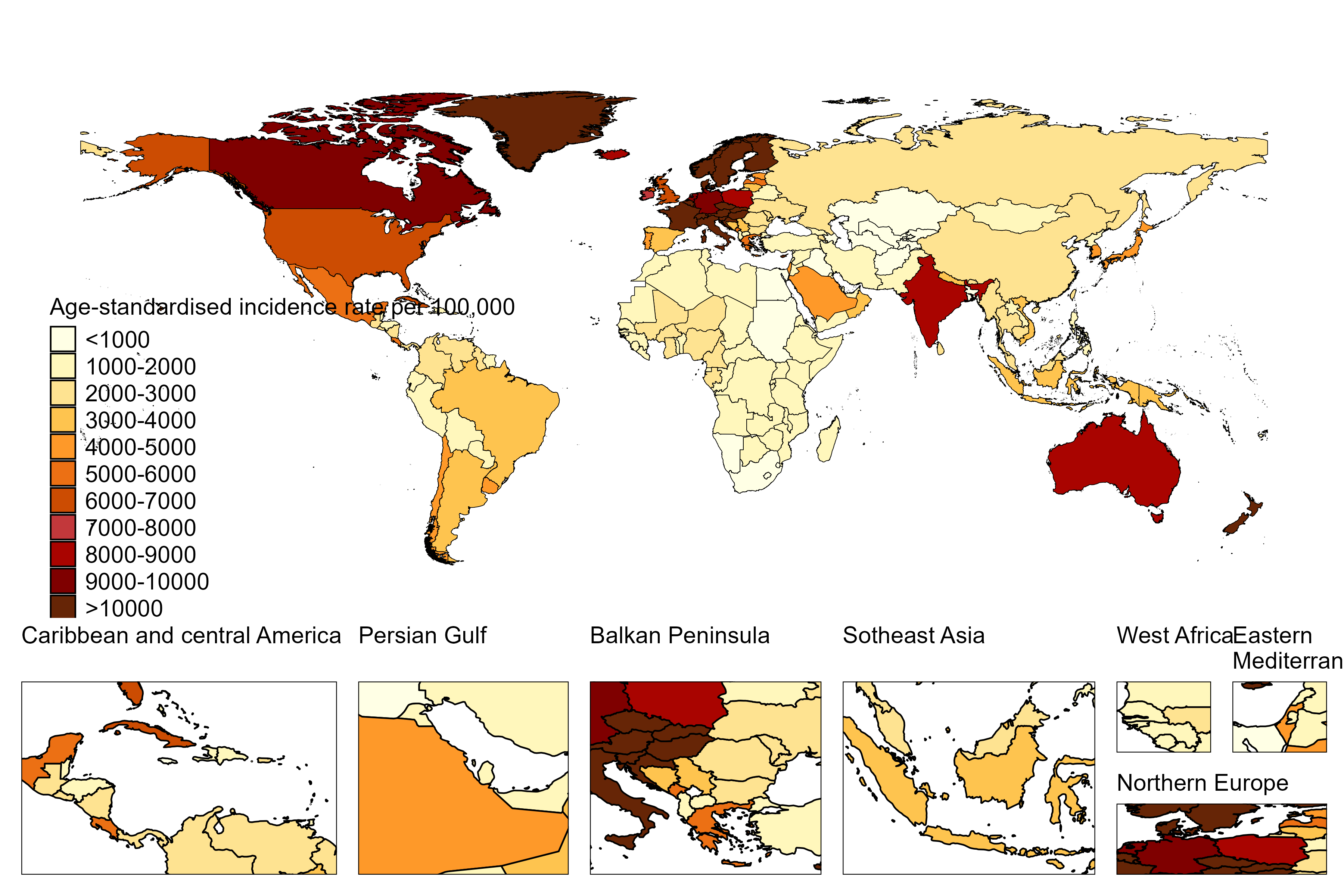 |
| --- |
| (a) |
| 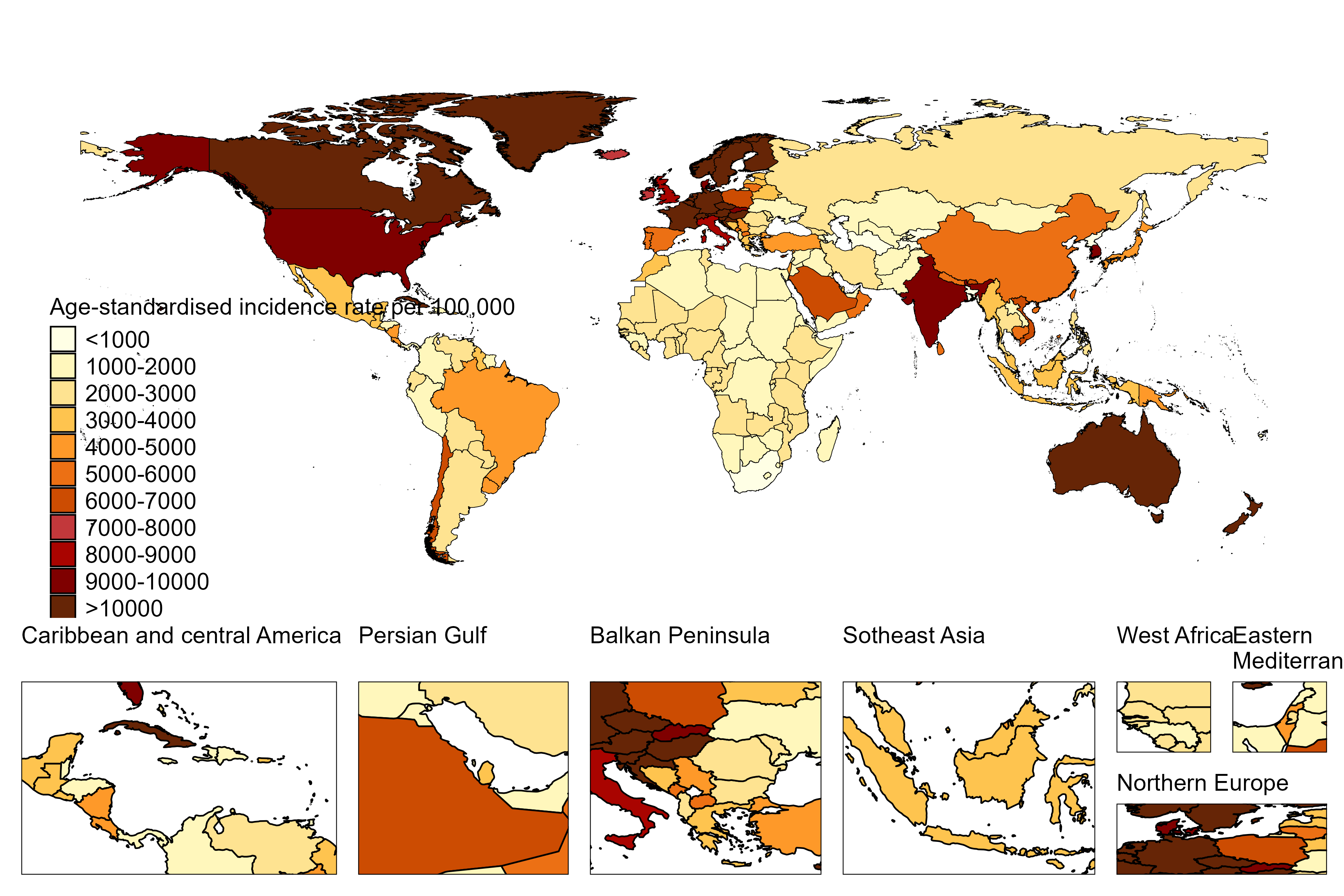 |
| (b) |
| 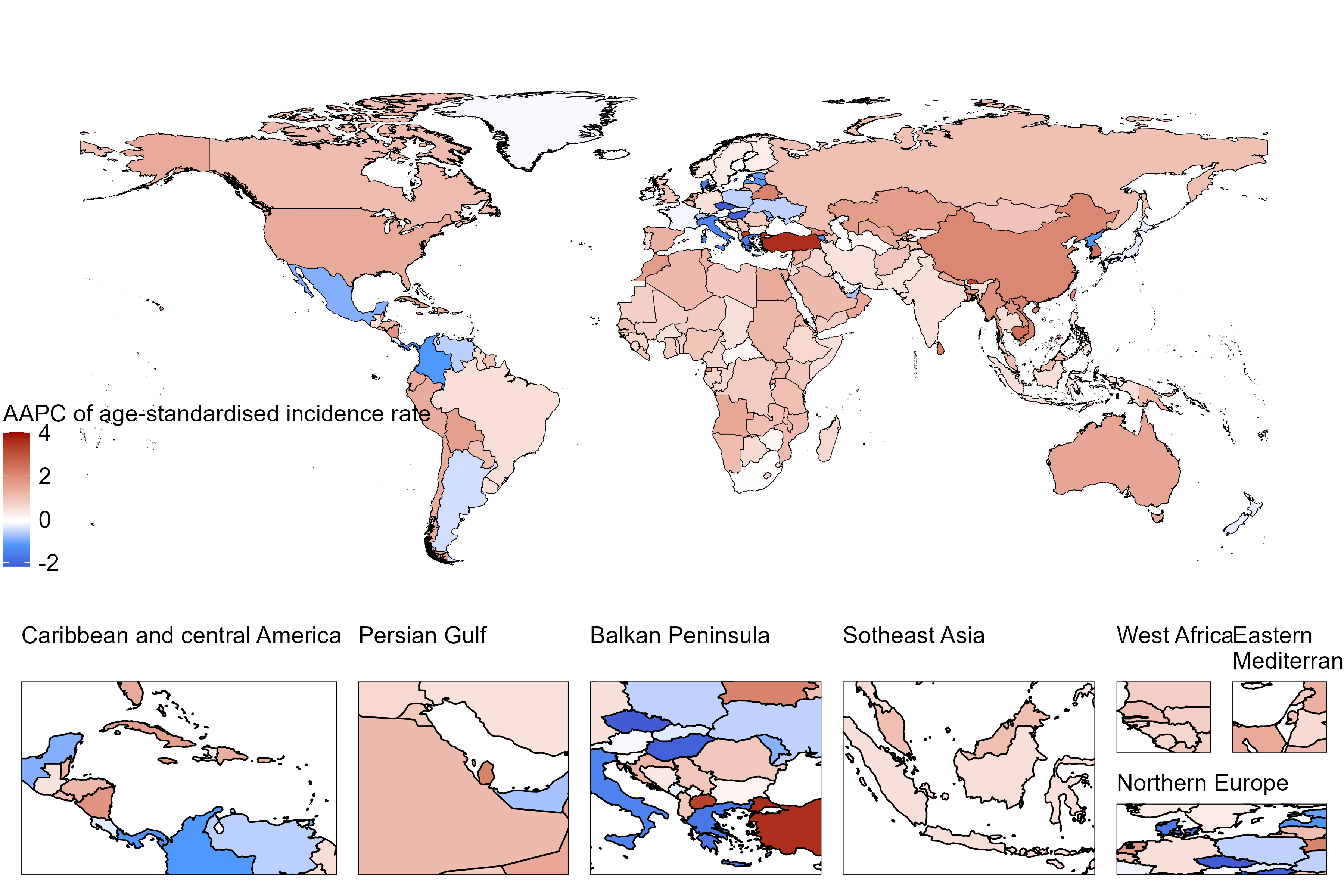 |
| (c) |
| Figure S1. Spatiotemporal patterns of incidence: (a) ASR of incidence in 1990; (b) ASR of incidence in 2021; (c) AAPC of ASR of incidence from 1990 to 2021. |

| 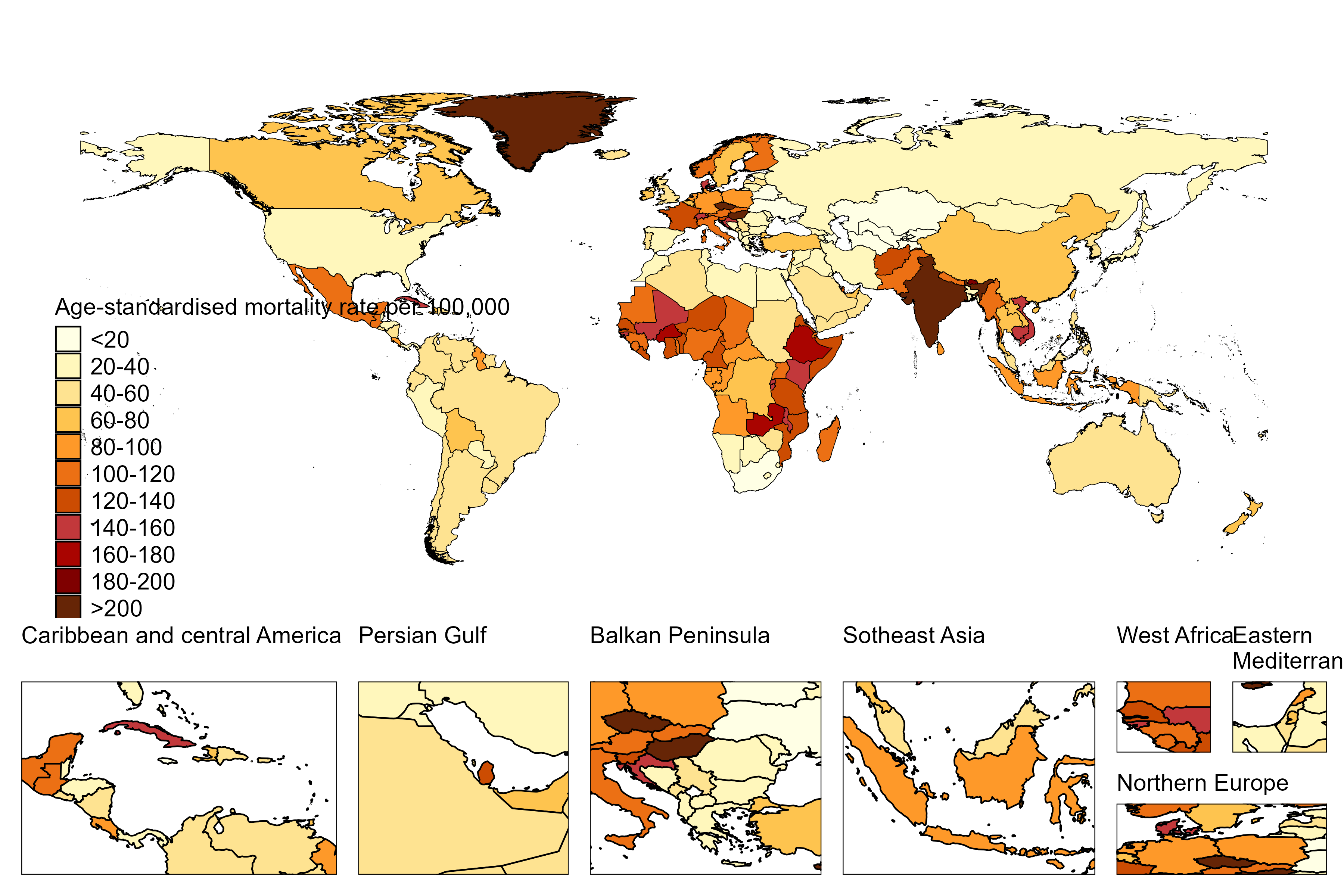 |
| --- |
| (a) |
| 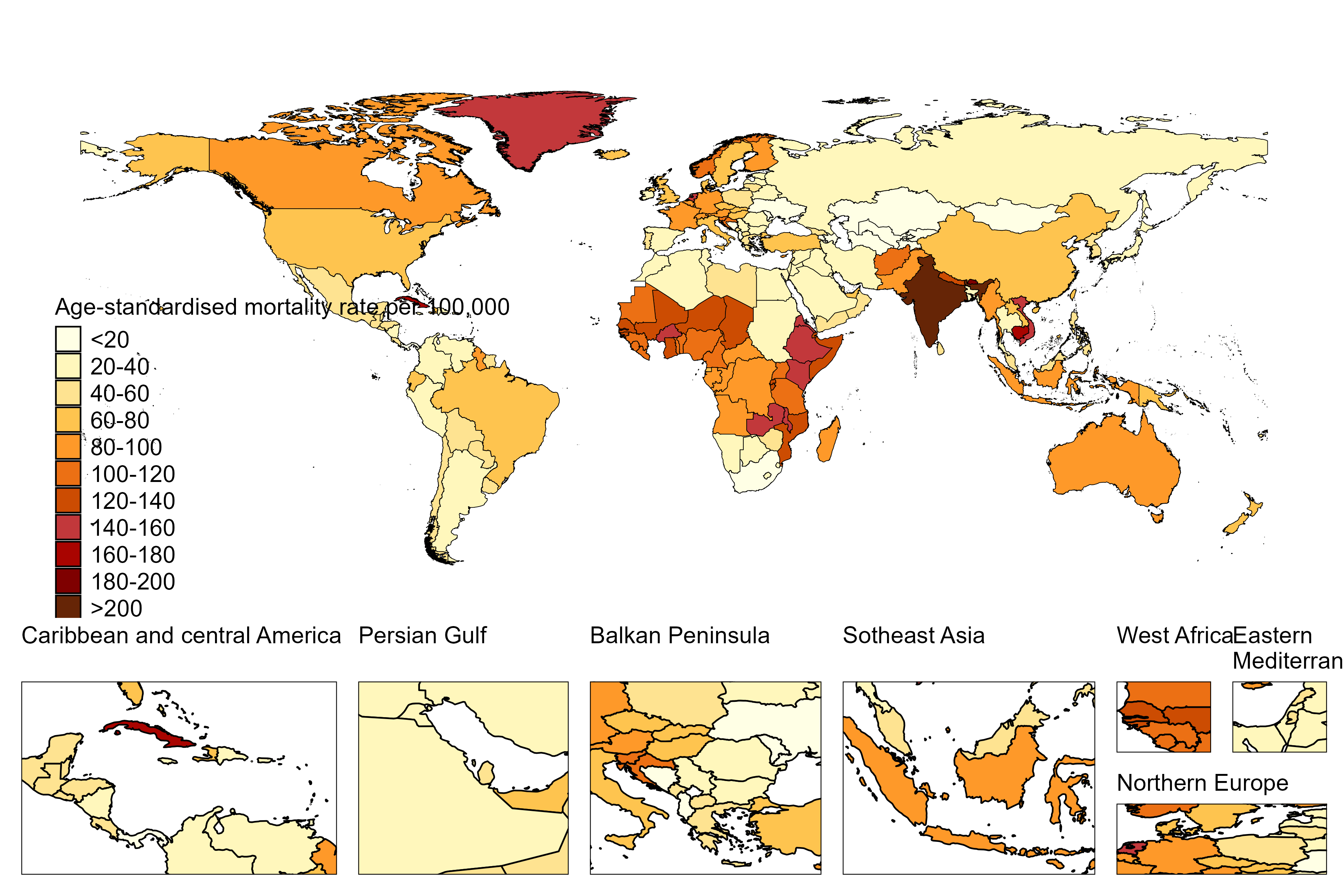 |
| (b) |
| 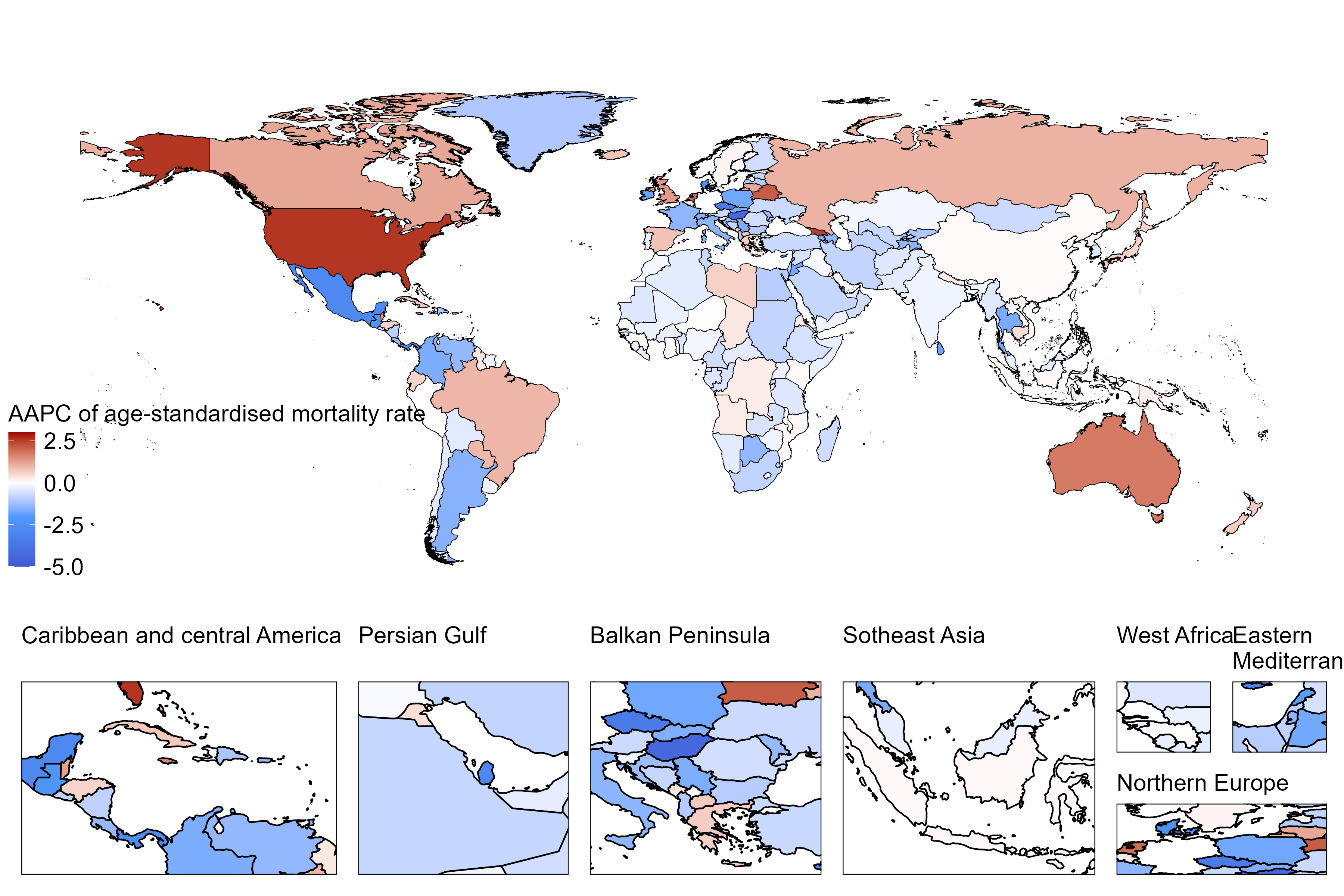 |
| (c) |
| Figure S2. Spatiotemporal patterns of mortality: (a) ASR of mortality in 1990; (b) ASR of mortality in 2021; (c) AAPC of ASR of mortality from 1990 to 2021. |

| 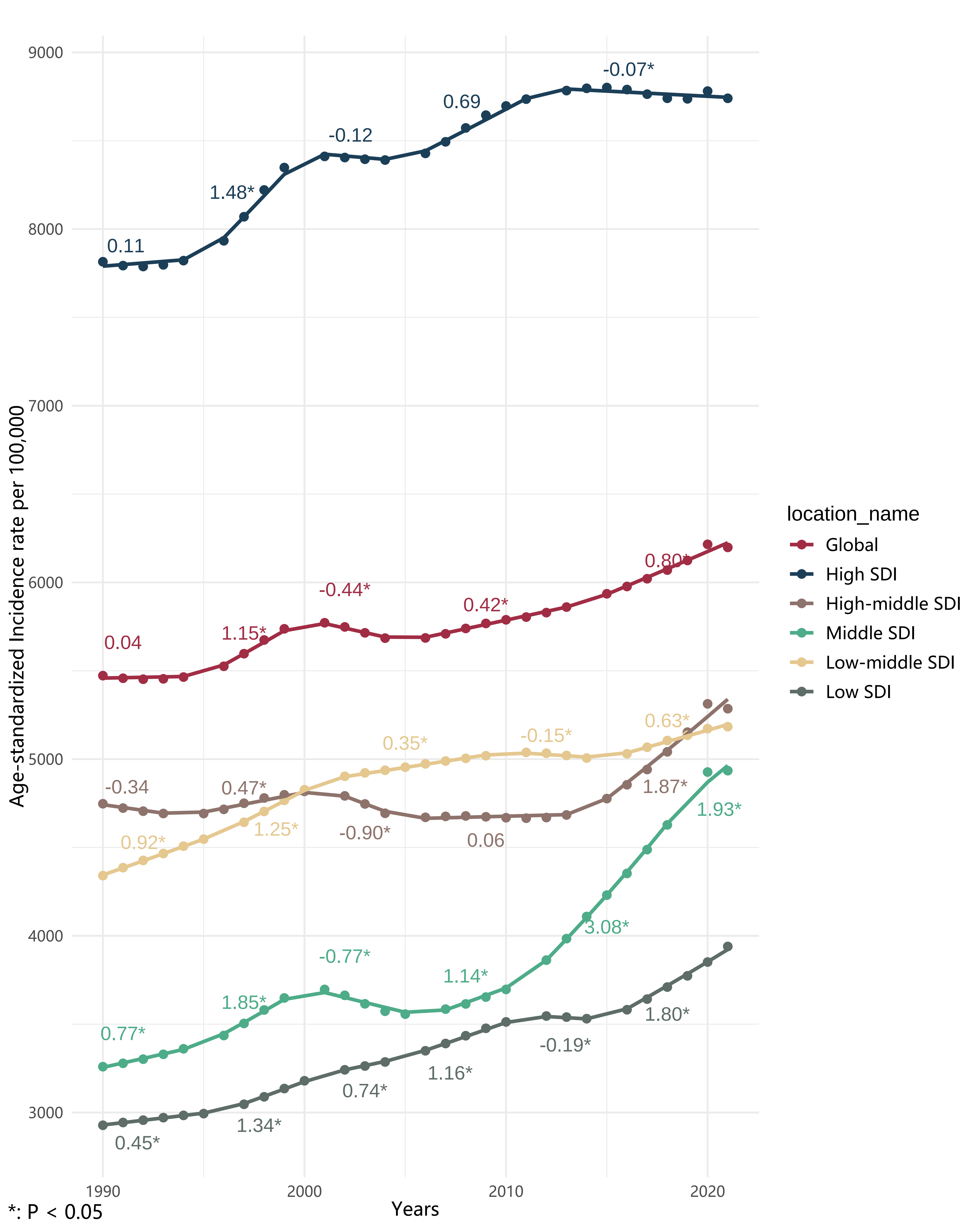 | 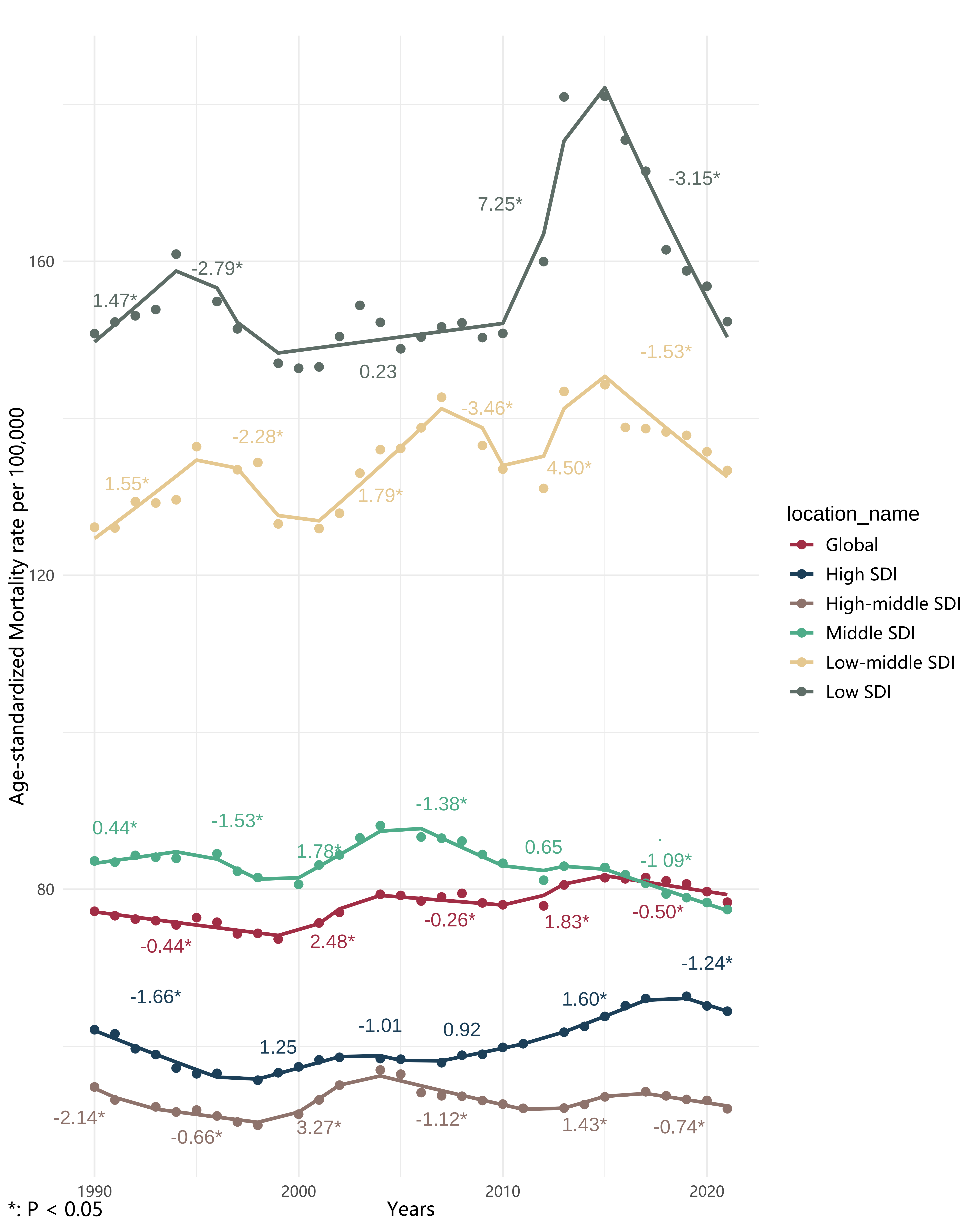 |
| --- | --- |
| (a) | (b) |
| Figure S3. Joinpoint regression analysis: (a) ASRs of incidence; (b) ASRs of mortality. | |
